# Accumulation of endogenous free radicals is required to induce titan-like cell formation in *Cryptococcus neoformans*

**DOI:** 10.1128/mbio.02549-23

**Published:** 2023-12-11

**Authors:** Irene García-Barbazán, Alba Torres-Cano, Rocío García-Rodas, Martin Sachse, Daniel Luque, Diego Megías, Oscar Zaragoza

**Affiliations:** 1 Mycology Reference Laboratory, National Centre for Microbiology, Instituto de Salud Carlos III, Majadahonda, Madrid, Spain; 2 Electron Microscopy Unit, Central Core Facilities, Instituto de Salud Carlos III, Madrid, Spain; 3 Advanced Optical Microscopy Unit, Central Core Facilities, Instituto de Salud Carlos III, Madrid, Spain; 4 Center for Biomedical Research in Network in Infectious Diseases (CIBERINFEC-CB21/13/00105), Carlos III Health Institute, Madrid, Spain; University of Minnesota Medical School, Minneapolis, Minnesota, USA

**Keywords:** *Cryptococcus neoformans*, off-patent drug repurposing, titan-like cells, reactive oxygen species, mitochondria

## Abstract

*Cryptococcus neoformans* is an encapsulated yeast able to cause disease (mainly meningoencephalitis) among immunosuppressed patients, mostly HIV+. This yeast can form the so-called titan cells *in vivo*, which are cells of an abnormally larger size due to an increase in both the capsule and the cell body size (total size reaching between 50–70 μm). This phenomenon can be partially reproduced *in vitro* to obtain cells of an intermediate size (25–30 μm), which have been denominated titan-like cells. In this work, we have screened 1,520 compounds from the Prestwick Chemical Library and identified off-patent drugs that inhibited titan-like cell formation *in vitro*. We developed an automated fluorescence-based microscopy assay and identified 64 compounds as possible inhibitors of titan-like cells. We chose 10 of these compounds to perform dose-response curves and confirmed them as inhibitors of titan-like cell formation. Several of the compounds identified had antioxidant properties (i.e., retinoic acid), indicating a possible role of free radicals during titan cell formation. Using fluorescent probes, we found that there was an endogenous accumulation of reactive oxygen species (ROS) during cell growth, which was inhibited in the presence of retinoic acid. Furthermore, we found that during titanization, there were significant changes in the mitochondria, which is the main organelle where ROS are produced. We hypothesize that an intracellular increase of free radicals at the mitochondria might be a triggering signal to induce titanization.

**IMPORTANCE:**

*Cryptococcus neoformans* is an excellent model to investigate fungal pathogenesis. This yeast can produce “titan cells,” which are cells of an abnormally larger size that contribute to the persistence of the yeast in the host. In this work, we have used a new approach to characterize them by identifying drugs that inhibit this process. We have used a repurposing off-patent drug library, combined with an automatic method to image and analyze fungal cell size. In this way, we have identified many compounds that inhibit this transition. Interestingly, several compounds were antioxidants, allowing us to confirm that endogenous ROS and mitochondrial changes are important for titan cell formation. This work provides new evidence of the mechanisms required for titanization. Furthermore, the future characterization of the inhibitory mechanisms of the identified compounds by the scientific community will contribute to better understand the role of titan cells in virulence.

## INTRODUCTION

*Cryptococcus neoformans* is a pathogenic yeast widely distributed in the environment that can cause disease in humans (1). Nowadays, it is responsible for hundreds of thousands of deaths each year, particularly in developing areas (2, 3). Cryptococcosis affects mainly immunocompromised patients (2, 3). Infection is initiated by inhalation of infective particles, which can colonize the lungs (4–6). Immunocompetent hosts control the infection, but in people with weakened immune system, the yeasts can disseminate to the central nervous system, causing meningoencephalitis (7).

*Cryptococcus neoformans* is a unique fungal pathogen due to its ability to adapt to different environments and infect different hosts (8–17). There are several reasons that explain this unique adaptation among fungal pathogens, such as its ability to melanize (18, 19) or to survive within phagocytic cells (20–23). However, one of the most important aspects is the presence of a polysaccharide capsule around the cell body that interferes with the host immune response (24–33).

Another striking characteristic of this pathogen during infection is its ability to increase its cell size (including both the capsule and the cell body), producing what has been denominated as titan cells (TC) (34–37). Titan cells have been described as those cells with a cell body above 15 μm or with a total cell size (including capsule) above 30 μm (38). These cells are uninuclear polyploid cells with a big vacuole, a thick cell wall, and a dense capsule, and their formation *in vivo* depends on signaling pathways and receptors such as PKA and Gpr4, respectively (36, 37). Due to their size, they can persist for longer periods in the host, and their presence inhibits the phagocytosis of cells of regular size (39). The *in vivo* factors that trigger titan cell formation are poorly characterized, but a high proportion of titan cells have been found in mice with a Th2-polarized response or during asymptomatic infections (36, 40). The process of titanization can be partially reproduced *in vitro* by incubating at different conditions, including low-nutrient media supplemented with serum and a CO_2_-enriched atmosphere with oxygen limitation (41–43). This results in the appearance of cells of an intermediate size (around 15–25 μm), which had been denominated as titan-like cells. The possibility to mimic the titan cells *in vitro* has allowed unravelling several signaling pathways and genes required for titan cell formation (41–43). However, the exact molecular mechanisms involved in this process remain unknown.

In this work, we present a different strategy to identify new pathways and processes involved in titan-like cell formation. We have performed a screening of the Prestwick Chemical Library to identify off-patent compounds that pharmacologically inhibit titan-like cell development, which could provide new insights on the titanization process. We found drugs that blocked titanization in *C. neoformans* and noticed that several inhibitory drugs had antioxidant properties. We hypothesized that an endogenous accumulation of free radicals in the cell might be one of the signals that triggers titan cell formation in *C. neoformans*.

## MATERIALS AND METHODS

### Prestwick Chemical Library and selected compounds

The Prestwick Chemical Library (Prestwick Chemical Libraries, GreenPharma, Strasbourg, Illkirch, France) was used to screen compounds that inhibit the formation of titan-like cells in *C. neoformans*. The library contains 1,520 off-patent compounds approved by different agencies such as the Food and Drug Administration and the European Medicines Agency. The Prestwick Chemical Library is prepared in 96-well plates, each well containing a different compound at 10 mM in 100% dimethyl sulfoxide (DMSO). Columns 1 and 12 in each plate are empty without compounds. For some experiments, we additionally used the antioxidants N-acetyl cysteine (Sigma Aldrich) and ascorbic acid (Sigma Aldrich).

### Yeast strains and growth conditions

*Cryptococcus neoformans* H99 strain [var. grubii, serotype A (44)] was used in most experiments. For specific experiments, *Cryptococcus deneoformans* B3501 (45) and *Cryptococcus deuterogatti* R265 (46) strains were also used. Yeasts were routinely grown in Sabouraud liquid or solid medium (Oxoid) at 30°C. For the induction of titan-like cells, we followed the protocol described in reference (43). Briefly, the yeasts were grown overnight in Sabouraud liquid medium at 30°C with moderate shaking at 150 rpm. The cells were then washed twice with 50 mM 3-(N-morpholino)propanesulfonic acid (MOPS, Sigma Aldrich) and suspended at 2 × 10^4^ cells/mL in titan cell medium (TCM), composed of 5% Sabouraud, 5% inactivated fetal calf serum (FCS, Biological Industries), and 15 µM sodium azide (Sigma Aldrich) and diluted in 50 mM MOPS adjusted to pH 7.3. Cells were incubated for 16 h at 37°C with a 5% CO_2_-enriched atmosphere.

### Growth assays

In some experiments, titan-like cells were induced as described above in TCM in the presence of different DMSO (Sigma Aldrich) concentrations (ranging from 2% to 0.03%, twofold serial dilutions). Photographs were taken using a Leica DMI3000 microscope (Leica Microsystems) with a Leica DFC 300 FX digital camera and LAS AF software (Leica Microsystems). Cell size was measured with Image J software (http://rsb.info.nih.gov/ij). Growth was also monitored in the presence of different percentages of DMSO at a Multiskan FC spectrophotometer (Thermo Fisher Scientific) by incubating at 37°C and measuring the optical density (OD) at 540 nm every 60 minutes during 48 h. Results were analyzed using GraphPad Prism software, version 9.0. As control, cells without DMSO were carried out in parallel in both experiments.

### Screening protocol and automated fluorescence-based microscopy protocol

To evaluate the effect of the compounds of the chemical library on titan-like cell formation, we first performed a 1/10 dilution of the compounds to obtain intermediate plates at 1 mM and 10% DMSO in 96-well round bottom plates (Falcon). Five microliters from these intermediate plates were diluted 20 times in sterile distilled water to obtain 2× stocks of the compounds (0.05 mM in 0.5% DMSO in a final volume of 100 µL) in 96-well plates with flat glass bottom (Greiner). In parallel, cells incubated overnight (o.n.) in Sabouraud liquid medium as described above were prepared at 2 × 10^4^ cells/mL in 2× TCM, and 100 µL was added to each well containing the compounds. In this way, the final screening was performed in 1× TCM with a cell density of 10^4^ cells/mL with the compounds at 25 µM and 0.25% DMSO. The following controls were added to columns 1 and 12 (four wells of each control in each plate): (i) cells grown in 1× TCM, (ii) cells grown in 1× TCM without serum, (iii) cells grown in sterile distilled water, and (iv) cells grown in Sabouraud liquid medium. All the controls contained DMSO 0.25%.

The plates were incubated for 16 h at 37°C with a 5% CO_2_-enriched atmosphere. After this incubation, all of the wells were visually observed with a DMI3000 microscope (Leica microsystems) to identify inhibitory compounds of titanization. In addition, we also developed an automated protocol that allowed us to take pictures of all the wells and measure the cell size in the presence of all the compounds. We used lactofuchsin, a dye that binds to the surface of fungi. This dye provides a light red staining of the cells, which is strongly fluorescent and easily observed in fluorescence microscopes using the standard rhodamine filters. A lactofuchsin stock was prepared with acid fuchsin (Sigma) at 1 mg/mL in 63% of lactic acid (Merck), and from this stock, we made a 1/30 dilution in distilled water (33 µg/mL in 2.1% of lactic acid). Finally, 30 µL from this dilution was added to each of the wells for the screening, so the final lactofuchsin concentration in each well was 4.3 µg/mL with lactic acid at 0.27%.

After staining, the cells were directly observed with a Cytell automatic microscope (GE Healthcare Life Sciences) using the 10× objective. Five pictures from each well were taken in bright field and with the rhodamine fluorescence filter. In total, 480 images were obtained for each of the 96-well plates. The images were analyzed in two different automatic ways. First, we used the analysis options from the Cytell software, so a dot plot representing the size of the cells detected and the fluorescence intensity was obtained for each well. Second, the 480 images were exported in TIF format (16 bits) and analyzed using Fiji software (47) using the batch mode option, with an in-house designed macro. Briefly, this macro creates first a mask of the fluorescent cells, and then, the Feret diameter and area of each cell identified in the mask are determined. In this way, around 250–500 cells per well are automatically measured. Results were exported as a .csv document, which was further processed with Microsoft Excel program using the PivotTable option, obtaining the area, average diameter, and standard deviation of the cells in the presence of each compound.

### Dose-response curves of selected compounds for confirmation

Selected compounds were bought as powder (Prestwick Chemical Libraries, GreenPharma, Strasbourg, Illkirch, France) and dissolved in 100% DMSO to a concentration of 50 mM. An intermediate stock of each compound was prepared at 20 mM in 50% DMSO. Dose-response experiments were performed in TCM, starting with a compound concentration of 100 µM and 0.25% DMSO and carrying out 11 twofold dilutions in 0.25% DMSO. A control in TCM without any compound and 0.25% DMSO was always added to the assay. The plates were incubated at 37°C in the presence of 5% CO_2_ as described above. Cell size was determined after staining with lactofuchsin, and analysis with the Cytell microscope was performed following the automatic pipeline described previously.

### Viability assays

Titan cells were induced by incubating yeast in TCM at 5 × 10^4^ cells/mL in the presence of different concentrations of the selected compounds. A control without any compound was always carried out. Different dilutions were made, and 100 µL of the cells at times 0 and 24 h was placed on agar Sabouraud plates. Plates were incubated at 30°C for 48 h, and the number of colony-forming units (CFUs) was determined. Two biological replicates were done, with three technical replicates each time, plating each condition in duplicate. To calculate the viability percentage, the number of CFUs in each condition was normalized by the number of CFUs obtained at time 0 without any compound.

### Endogenous reactive oxygen species detection by flow cytometry

To detect endogenous reactive oxygen species (ROS), we used the ROS-susceptible probe dihydrofluorescein diacetate (DHF, Sigma-Aldrich), prepared at a stock concentration of 4 mM in 100% DMSO. When this probe reacts with ROS, it produces fluorescein molecules, providing green fluorescence. Yeasts were cultured overnight in Sabouraud liquid medium as described previously, washed with PBS, and suspended at 5 × 10^4^ cells/mL in Sabouraud liquid medium or TCM. Cells were incubated in 12-well tissue culture plates (Falcon) at 37°C with a 5% CO_2_-enriched atmosphere for different times (0, 3, 6, and 24 h). At each time, cells were stained with 40 µM DHF (1/100 dilution from initial stock) for the detection of endogenous reactive oxygen species. The cells were incubated for 30 minutes at 37°C with 5% CO_2_ and then washed twice with phosphate-buffered saline (PBS). For each time, a control sample without DHF was carried out. Fluorescence intensity measurement was done in a BD Accuri C6 Plus cytometer (BD Biosciences), counting 10,000 events. The data were analyzed with FlowJo v10 software (Tree Star, Inc.). As a control for DHF activity, cells were also treated at time 0 h with 1 µg/mL of amphotericin B for 1 h at 37°C with 5% CO_2_. Then, DHF was added, and fluorescence was detected as described above.

In some experiments, retinoic acid (25 µM) was added during the incubation of TCM, and ROS were detected as described above.

To normalize the amount of ROS produced by the size of each cell, we exported the original cytometry data from FlowJo into a .csv document containing all the intensity data from all the parameters. Then, we filtered the data eliminating those rows whose forward scatter (FSC) values were below 50. Then, we calculated the FL1/FSC × 10,000 ratio and exported the data to GraphPad Prism 9.0 to generate the graphs, geometric means, and statistics.

### Study of mitochondrial membrane potential by JC-1

The MitoProbe JC-1 Assay Kit [5′,6,6′-tetrachloro-1,1′,3,3′-tetraethylbenzimidazolylcarbocyanine iodide (JC-1, Life Technologies)] was used for the study of mitochondrial membrane potential. A stock at 200 µM was prepared in DMSO. This dye is accumulated in the mitochondria and produces green and red fluorescence depending on the membrane potential. When the mitochondria are functional, JC-1 emits both red and green fluorescence. In contrast, when the mitochondria are depolarized, this probe only emits green fluorescence. In this way, changes in red/green fluorescence ratio are indicators of variations in the mitochondrial membrane potential.

The cells were incubated at a cell density of 5 × 10^4^ cells/mL in Sabouraud liquid medium and TCM at 37°C with a 5% CO_2_-enriched atmosphere as described in the previous sections. After different incubation times (0, 3, 6, and 24 h), cultures were washed twice with PBS and suspended at 5 × 10^4^ cells/mL, and JC-1 was added at a final concentration of 2 µM. The samples were incubated at 37°C and 5% CO_2_, and fluorescence intensity of the cells was measured by flow cytometry using a BD Accuri C6 Plus cytometer (FL-1 channel for green fluorescence and FL-2 channel for red fluorescence). Data were analyzed with FlowJo v10 software (Tree Star, Inc.). To determine the red/green fluorescence ratio, we exported the raw fluorescence data in FlowJo to a .csv document which was processed with Excel. After setting a threshold of 50 to exclude unlabeled events, the ratio FL2 (red)/FL1 (green) for each cell was calculated. Then, the data were analyzed with GraphPad Prism 9.0, and the geometric mean for each population was calculated.

### MitoTracker staining

Cells were cultivated overnight and then incubated in Sabouraud liquid medium and TCM at 37°C and 5% CO_2_ as described in the previous sections. The cells were incubated for different times (0, 3, 6, and 24 h), and at each time point, a sample of the culture was collected, washed with PBS, and suspended at 5 × 10^4^ cells/mL. MitoTracker Red CMXRos (Invitrogen) was prepared at 1 mM in 100% DMSO, and then, an intermediate stock of 40 µM in PBS was freshly prepared. Finally, MitoTracker was added at 40 nM to the cultures and incubated for 30 minutes at 37°C with a 5% CO_2_-enriched atmosphere. Samples were washed twice with PBS, and the red fluorescence of the cells was imaged in different z-stacks in a Stellaris confocal microscope (Leica Microsystems). An image corresponding to the maximal projection of the fluorescence from all the z-stacks was generated with LAS X software (Leica Microsystems) and exported and processed with Fiji and Adobe Photoshop CS3.

### Measurement of oxygen consumption rate

Basal oxygen consumption rate of regular and titan-like cells was measured at different time points using a Seahorse XFe24 Analyzer (Agilent). The day before the run, the Seahorse Analyzer was turned on overnight to warm up, and the sensor cartridge was hydrated with 200 µL of distilled H_2_O and placed overnight at 37°C. Additionally, yeast cells were incubated overnight in Sabouraud liquid medium at 30°C with moderate shaking (150 rpm). On the day of the experiment, 200 µL of XF Seahorse calibrant solution was added to the utility plate to hydrate the sensor cartridge. Cells were incubated at 37°C and 5% CO_2_ in different conditions: TCM, TCM with 25 µM retinoic acid, and Sabouraud liquid medium (control). At the different time points (0, 3, 6, and 24 h), cultures were washed three times with Dulbecco's Modified Eagle's Medium (DMEM) and prepared at different cell densities to have a final homogeneous layer of yeasts at the microplates. One hundred eighty microliters of the corresponding fungal culture were added to each well of the microplate and placed into a 37°C incubator. Two wells were left without yeasts and just with DMEM assay medium as background control. Results were normalized by 100,000 cells.

### Transmission electron microscopy and quantification of the mitochondria

*C. neoformans* H99 cells were grown overnight as previously described. Cells were cultured to a final density of 5 × 10^4^ cells/mL in Sabouraud liquid medium for regular cells and in TCM for titan-like cells and incubated for 16 h at 37°C with a 5% CO_2_-enriched atmosphere. Both samples were pelleted down at 3,250 *× g*. The pellet (approximately 30-µL pellet) was washed twice with PBS and gently suspended in 2.5% paraformaldehyde + 0.1% glutaraldehyde in 0.1 M phosphate buffer, pH 7.2. After fixation for 2 h at room temperature (RT), the cells were washed with PBS, and the remaining free aldehydes were quenched with 50 mM NH_4_Cl in PBS. The cell pellets were embedded in 12% gelatine in PBS and solidified on ice. Next, cubes of 1 mm^3^ were cut and infiltrated with 2.3 M sucrose in PBS overnight at 4°C. The cubes were mounted on metal pins and frozen by plunging into liquid nitrogen. Thin sections were cut with a cryo-microtome UC7 (Leica Microsystems) with a nominal thickness of 70 nm and picked up with a 1:2 mixture of 2% methylcellulose in water and 2.3 M sucrose in PBS. After thawing, the sections were deposited on 100 mesh copper grids with a carbon formvar film. To contrast the grids, they were incubated on PBS for 30 minutes at 37°C, washed extensively with water, and incubated for 5 minutes on ice with 0.4% uranyl acetate in 1.8% methylcellulose in water. Excess of contrasting solution was removed by blotting the grids with filter paper. Images were taken in a Tecnai G2 microscope operated at 120 kV with a Ceta camera.

To measure the surface area of the mitochondria on thin sections, images of all mitochondria from 10 cell profiles were taken at a nominal magnification of 30,000×. The surface area of mitochondria was measured with Fiji software using the freehand selection tool. The number of cristae was counted to calculate the number of cristae per surface area for each mitochondrion. In total, 58 mitochondria were analyzed for the control cells, and 106, for titan-like cells.

### Statistical analysis

Normality of the samples was assessed by the Kolgomorov-Smirnov test. When the samples presented a Gaussian distribution, statistical differences of the cell sizes were assessed using analysis of variance with the Bonferroni post-test (significance when *P* < 0.05). If the samples were not normally distributed, the Kruskal-Wallis test with the Dunn’s post-test was used. In all cases, the degree of significance (*P* < 0.05; *P* < 0.0001) is indicated. All calculations were performed using GraphPad Prism 9.0.

## RESULTS

### Determination of the optimal DMSO concentration at which DMSO did not inhibit the formation of titan-like cells

**Fig 1:**
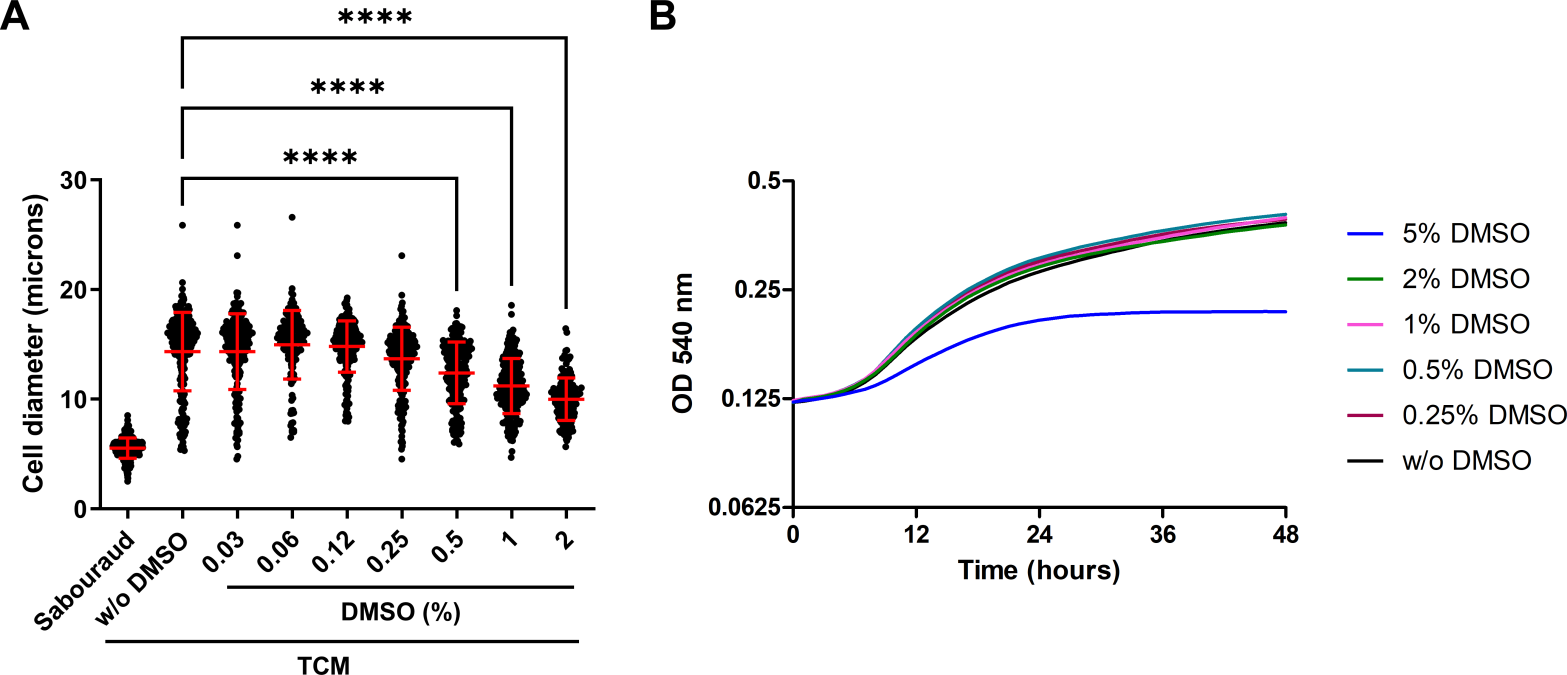
Effect of DMSO in *C. neoformans* H99 strain. **(A**) Effect of DMSO in the formation of titan-like cells. A dose-response curve in TCM with different concentrations of DMSO was performed to determine the maximum percentage of DMSO to be used during the screening of the Prestwick Chemical Library. Cells in Sabouraud liquid medium were added as control of regular size cells. Asterisks denote statistical differences (*P* < 0.0001, Kruskal-Wallis test), and the red bars denote the average and the standard deviation of the population. (**B**) Growth curves of H99 strain in Sabouraud liquid medium at 37°C in the presence of different percentages of DMSO. Growth curves were obtained during 48 h by measuring the OD at 540 nm every 60 minutes.

The compounds of the Prestwick Chemical Library were prepared at a concentration of 10 mM in 100% DMSO, so we first evaluated the effect of this solvent on titan-like cell formation. For this purpose, we performed a dose-response curve to determine the maximum DMSO concentration that did not interfere with this process. As shown in Fig. 1A, *C. neoformans* significantly enlarged in size at all the DMSO concentrations tested. However, we found that at concentrations between 0.5% and 2%, there was a partial inhibition of cell growth. As a consequence, we decided that the final concentration of DMSO should not exceed 0.25% during titan-like cell induction. We also performed growth curves in the presence of different concentrations of DMSO (5%–0.25%). As shown in Fig. 1B, no inhibition of cell growth was observed with 0.25%–2% of DMSO, and only a higher percentage of DMSO (5%) caused a reduction of the growth rate.

### Standardization of a fluorescence-based protocol to visualize titan-like cells

**Fig 2:**
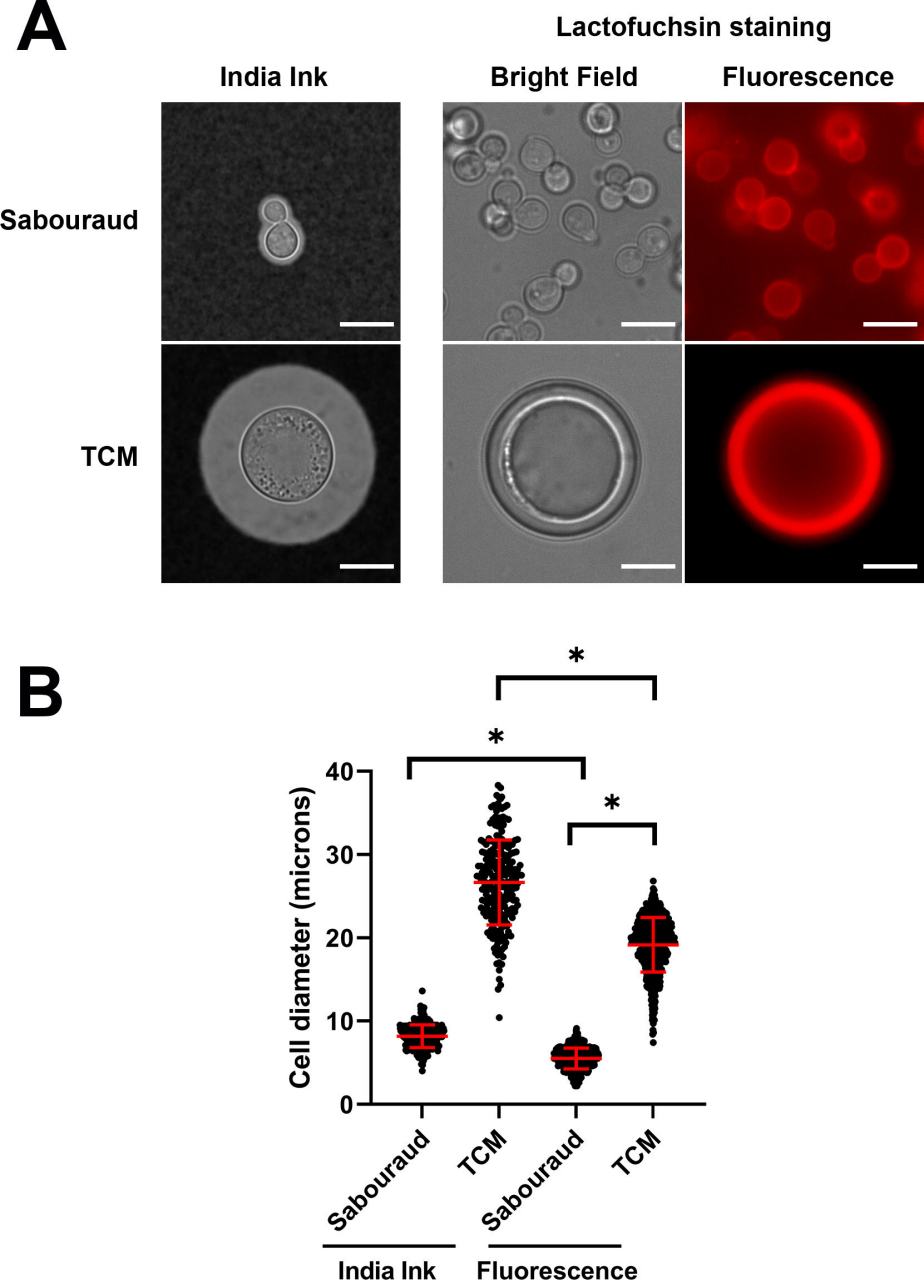
Sizes of *C. neoformans* measured after suspension in India ink or with lactofuchsin staining. (**A**) Morphology of the cells grown in Sabouraud liquid medium (upper panels) and TCM (lower panels) with different stainings, India ink (right) and lactofuchsin (left). Scale bar in every panel corresponds to 10 µm. (**B**) Differences in size measured after suspension in India ink or staining with lactofuchsin. Asterisks show statistical difference (*P* < 0.0001, *t*-test), and the red bars denote the average and the standard deviation of the population.

We next wanted to describe a protocol that allowed us to visualize the size of the cells based on fluorescence, since this eases further automatic analysis of the images compared to bright field pictures. Our laboratory has employed other dyes to better visualize fungal structures, with lactofuchsin being one of the most used. Lactofuchsin binds non-specifically to the cell wall of fungi, allowing its visualization with a light red-pink color. We observed that it also binds to the surface of *C. neoformans*, providing a strong fluorescence using standard rhodamine fluorescence cubes (Fig. 2). For this reason, we investigated if lactofuchsin was suitable to visualize the whole size of the cells, including the capsule.

At concentrations around 5 µg/mL, we observed a clear and bright fluorescence staining of titan-like and regular cells, including the capsule. In the case of titan-like cells, this staining was also visible in bright field due to a Quellung effect-like phenomenon (see Fig. 2A). Then, we confirmed that the staining with lactofuchsin did not alter the regular cell size measured by other standard protocols. As shown in Fig. 2B, the size of the cells detected by lactofuchsin fluorescence was slightly smaller to that measured after suspending the cells in India ink. This was mainly due to an increase in capsular packing and was even observed when the cells were suspended in lactic acid, the solvent in which fuchsin is dissolved (data not shown). Despite this difference, lactofuchsin staining still clearly differentiated titan-like cells from control cells incubated in non-inducing medium.

### Screening for compounds that inhibit the formation of titan-like cells in *Cryptococcus neoformans*

To identify compounds that inhibited titan-like cell formation, we used the Prestwick Chemical Library, which contains 1,520 off-patent drugs and has been used in repurposing experiments. The compounds in this library are dissolved at 10 mM in 100% DMSO. To avoid the small inhibitory effect of DMSO on titan cell formation at concentrations above 0.5%, for the screening, we chose a concentration of 25 µM (1/400 dilution of the original chemical library) for each drug (0.25% DMSO concentration).

For the screening, plates with TCM and individual compounds were prepared as described in Materials and Methods and inoculated at 10^4^ cells/mL. After an o.n. incubation, the plates were visualized under the microscope to identify wells in which the cells had not grown in size. In addition, we stained them with lactofuchsin, and the cell size was measured with a Cytell microscope and an in-house developed pipeline that identified and measured all the cell sizes from all the images (see Materials and Methods).

We identified 99 compounds that inhibited titan-like cell development. We categorized these compounds depending on the final size of the cells after the incubation in TCM (≤10 μm and >10 to ≤15 μm). Among these, we found several well-known antifungals, such as amphotericin B, voriconazole, itraconazole, fluconazole, nystatin, and terbinafine. We also found drugs that presented inhibitory activity against *C. neoformans* in previous screenings performed with the same library (48, 49). For further analysis of the results, we discarded these compounds, finally obtaining 64 potential drugs that inhibited titan-like cell formation. The identified compounds belonged to several therapeutic classes, including antimicrobials, endocrinology, metabolism, central nervous systems, dermatology, allergology, oncology, and rheumatology (see Table 1 for the complete list).

**TABLE 1:** List of compounds that inhibited TC formation

Control samples	Diameter (μm)	% titan cells	
Control in TCM	21.5	93	
Control in Sabouraud	8	0.5	

Compound	Diameter	% titan cells	Therapeutic class	Therapeutic effect	Target
Mitoxantrone dihydrochloride	5.8	0	Oncology	Antineoplastic	Topoisomerase II, DNA, and RNA
Alexidine dihydrochloride	6.7	0	Infectiology	Antibacterial	
Ebselen	6.7	0	Metabolism, central nervous system	Anti-inflammatory, anti-oxidant, antidiabetic, central nervous system stimulant	Bifunctional epoxide hydrolase 2, acetylcholinesterase
Ethacridine lactate	6.7	0	Infectiology, endocrinology	Antifungal, abortifacient	Cyclooxygenase-1
Thimerosal	6.8	0	Infectiology	Antiseptic, antifungal	Inositol 1,4,5-trisphosphate-sensitive calcium-release channel
Cinacalcet hydrochloride	6.8	0	Endocrinology	Antihyperparathyroid	Extracellular calcium-sensing receptor
Methyl benzethonium chloride	7.1	0	Infectiology	Antibacterial	
Merbromin	7.2	0	Infectiology	Antibacterial	
Propidium iodide	7.2	0	Infectiology	Antibacterial	
Pentamidine isethionate	7.3	0	Infectiology	Antifungal, antiparasitic, antiprotozoal	
Tolonium chloride	7.5	0	Cardiovascular, diagnostic	Hemostatic, diagnostic	DNA or RNA
Pyrvinium pamoate	7.7	0.7	Metabolism		
Flupentixol dihydrochloride cis-(Z)	8	0	Central nervous system	Central nervous system stimulant, antipsychotic	Dopamine D1 receptor
Nitroxoline	8.1	0	Infectiology	Antibacterial	Cyclooxygenase-2, cathepsin B, methionine aminopeptidase 2
Sertraline	8.2	1.3	Central nervous system	Antidepressant, central nervous system stimulant	Serotonin transporter
Clioquinol	8.6	0	Metabolism	Antiamebic, antifungal, antiseptic	
Broxyquinoline	9	0.4	Metabolism	Antidiarrheal, antiprotozoal	Acyl-CoA-binding protein
Methylene blue	9.2	0	Dermatology, hematology	Antiseptic, antidote, antimalarial, antiseptic	NADH-cytochrome b5 reductase 3, nitric oxide synthase
Chlorquinaldol	9.7	0	Infectiology	Antibacterial, antiseptic, antifungal	Bacteria growth
Proflavine hemisulfate	10	1.8	Infectiology, diagnostic	Antiseptic, antibacterial, contrastant	Bacterial RNA, DNA
Butenafine hydrochloride	10.3	1.2	Infectiology, metabolism	Antifungal	
Lomitapide	10.3	3.6	Metabolism	Hypocholesterolemic	Microsomal triglycerid transfer protein, neurokinin 1 (NK1) receptor
Ebastine	10.7	0.7	Allergology	Antihistaminic	Histamine H1 receptor
Sulconazole nitrate	10.8	1.3	Metabolism	Antifungal	
Econazole nitrate	11.1	6.5	Antifungal	Antifungal	
Halofantrine hydrochloride	11.2	7.0	Metabolism	Antimalarial	
Phenoxypropazine dihydrochloride	11.2	0.8	Central nervous system	Antidepressant	Monoamine oxidase
Butoconazole nitrate	11.3	14.4	Infectiology, metabolism	Antibacterial, antifungal	
Isotretinoin	11.4	11.0	Keratolytic		
Retinoic acid	11.4	10.1	Keratolytic		
Metyrapone	11.6	9.9	Endocrinology		
Enilconazole	11.9	8.1	Metabolism	Antifungal	
Liranaftate	12.1	18.2	Infectiology, metabolism	Antifungal	
Albendazole	12.2	25.3	Antihelmintic, antiparasitic		
Amorolfine hydrochloride	12.2	13.8	Infectiology, dermatology	Antifungal	
GBR 12909 dihydrochloride	12.5	20.7	Central nervous system	Antidepressant	
Antimycin A	12.7	17.3	Antibacterial, antifungal	Antibacterial, antifungal	
Oxiconazole nitrate	12.7	22.0	Infectiology, metabolism	Antifungal	
Prenylamine lactate	12.9	26.4	Cardiovascular, central nervous system	Antianginal, anxiolytic, vasodilator	
Tacrolimus monohydrate	12.9	5.3	Immunology	Immunosuppressant	Immunophilin FKBP-12, FK506-binding protein 1A
Flubendazol	13	44.0	Metabolism		
5-Fluorouracil	13	24.3	Oncology	Antineoplastic	Thymidylate synthase
Tinoridine hydrochloride	13	23.8	Rheumatology	Anti-inflammatory, analgesic, antipyretic	Lipid oxidation
Quinacrine dihydrochloride hydrate	13.2	28.9	Antihelmintic, antileishmanial, antimalarial, antiparasitic, antiprotozoal, antitrichomonal		
Zanamivir	13.2	24.9	Infectiology	Antiviral	Neuraminidase
Clotrimazole	13.5	25.9	Antibacterial, antifungal	Antibacterial, antifungal	
(S)-(-)-Cycloserine	13.5	28.3	Infectiology, metabolism	Antibacterial	
Verteporfin	13.5	41.1	Ophthalmology		
Carbazochrome	13.6	25.3	Hematology, dermatology	Hemostatic, anti-hemorrhoids	Alpha adrenergic receptor
Climbazole	13.7	25.7	Dermatology	Antifungal	Cytochrome P450
Terconazole	13.8	32.1	Infectiology, metabolism	Antifungal	
Exifone	13.8	39.7	Central nervous system	Central nervous system stimulant, nootropic	Anti-radical
Thiostrepton	13.9	34.7	Infectiology	Antibacterial	
Pinaverium bromide	14.1	33.5	Neuromuscular	Antispastic	
Clofazimine	14.2	36.5	Infectiology	Antibacterial	
Isoconazole	14.3	42.5	Infectiology, metabolism	Antibacterial, antifungal	
Cinepazide maleate	14.5	49.0	Cardiovascular	Vasodilator	Voltage-gated calcium channel
Phenelzine sulfate	14.8	51.4	Antidepressant	Enzyme	
Naftifine hydrochloride	14.8	49.6	Infectiology, metabolism	Antifungal	
1,8-Dihydroxyanthraquinone	14.9	49.6	Gastroenterology	Laxative, antiemetic	
Cisatracurium besylate	14.9	47.1	Neuromuscular	Muscle relaxant	

### Dose-response curve of selected compounds

**Fig 3:**
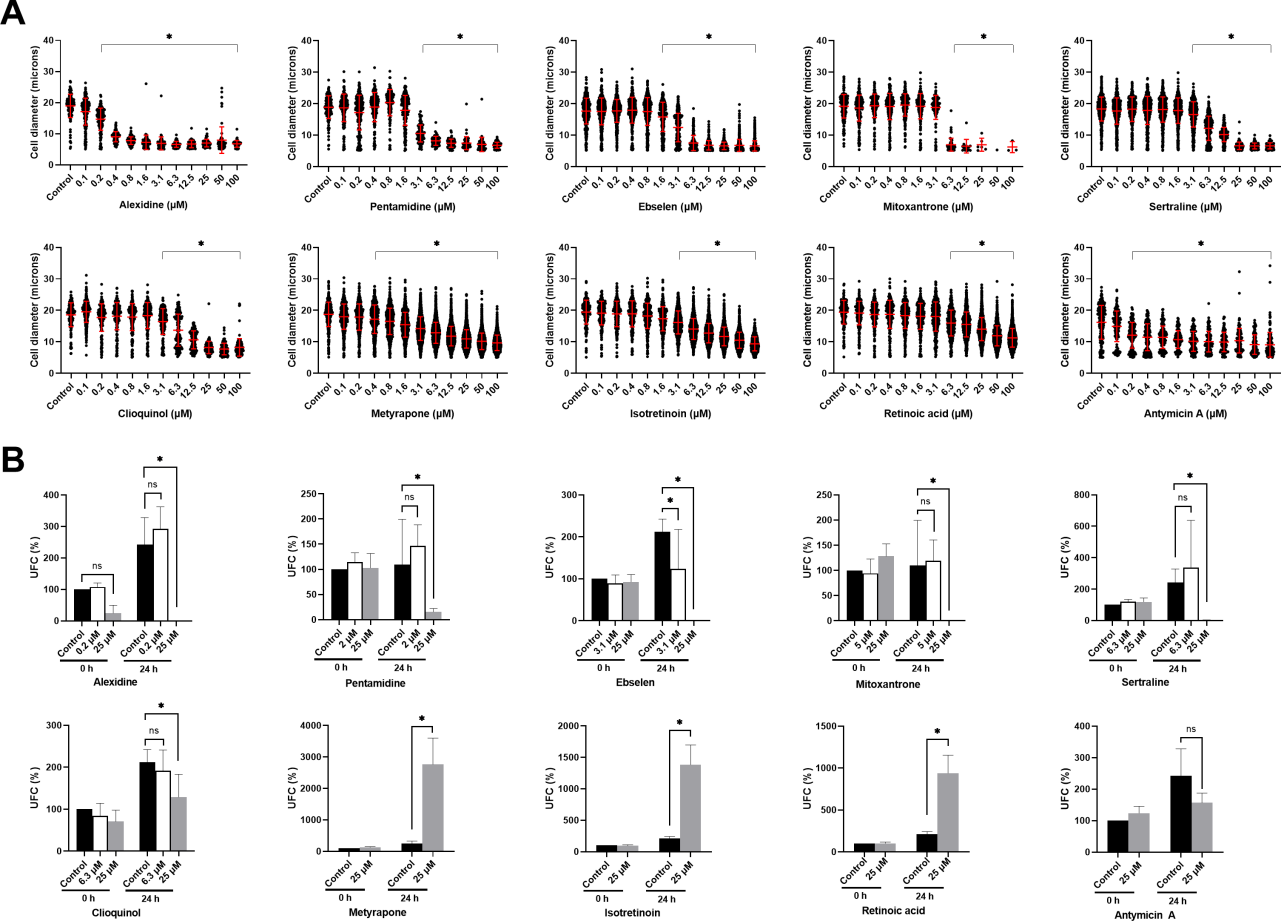
Effect of selected compounds on TC formation and viability. (**A**) Dose-response curves of the selected compounds. Cells were induced in TCM in the presence of different concentrations of each compound (ranging from 100 µM to 0.1 µM) as described in Materials and Methods. A control without any compound was added in all curves. Diameters of the cells were measured, and the average of the cell sizes and the standard deviations are represented in the figure. The experiment was performed in duplicate. Asterisks indicate *P* < 0.05 (Kruskal-Wallis test). (**B**) Effect of the selected compounds on the viability of the cells in TCM. Different concentrations of the selected compounds were analyzed to determine the inhibition of titan-like cells from each compound in *C. neoformans*. CFUs were determined at times 0 and 24 h. For each condition, we estimated the percentage of CFUs compared to the number of CFUs obtained at time 0 without exposure to any compound (see Materials and Methods). The average and standard deviations from two independent experiments with three different replicas are represented. Asterisk indicates *P* < 0.05.

To validate the results of the assay, we selected 10 compounds based on their mechanism of action and effect and performed dose-response experiments (from 100 to 0.1 µM, with a constant concentration of 0.25% of DMSO). These compounds were isotretinoin, retinoic acid, mitoxantrone dihydrochloride, pentamidine isethionate, alexidine dihydrochloride, clioquinol, metyrapone, sertraline, ebselen, and antimycin A. As shown in Fig. 3A, we confirmed that all selected compounds inhibited titan cell formation in a dose-dependent manner. Most of the drugs showed a lineal inhibition between 6 and 100 µM. In the case of mitoxantrone, concentrations around 6 µM fully inhibited cryptococcal cell growth. Alexidine dihydrochloride was the compound that showed the strongest inhibitory effect with concentrations below 1 µM, blocking almost completely the formation of titan cells.

All of the selected compounds inhibited the formation of titan-like cells but in different ways. The size of the cells obtained in each concentration varied for each compound. To determine if the inhibitory effect of the selected compounds on TC formation was due to possible fungicidal activities, we performed viability experiments by measuring the colony-forming units after incubating with the selected compounds. In these experiments, we chose different concentrations for each compound, one that caused an intermediate inhibition and another that resulted in the strongest reduction in TC formation (25 µM). Metyrapone, isotretinoin, retinoic acid, and antimycin A were only assayed at 25 µM, as the four of them had a similar profile. As shown in Fig. 3B, none of the compounds inhibited the viability of the yeasts after 24 h of exposure. The rest of the compounds were tested both at 25 µM and at an additional lower concentration. These compounds had a more drastic inhibitory profile, with a mean cell body diameter lower than 10 µm (Fig. 3A). The majority of the compounds produced a reduction in the number of CFUs at 25 µM, with the exception of clioquinol at 25 µM. However, at lower concentrations, except from ebselen, the rest of the compounds produced an inhibition in TC development without reducing the number of CFUs, indicating that at these concentrations, the inhibitory effect was not due to fungicidal effects.

### Effect of the retinoic acid in other strains

**Fig 4:**
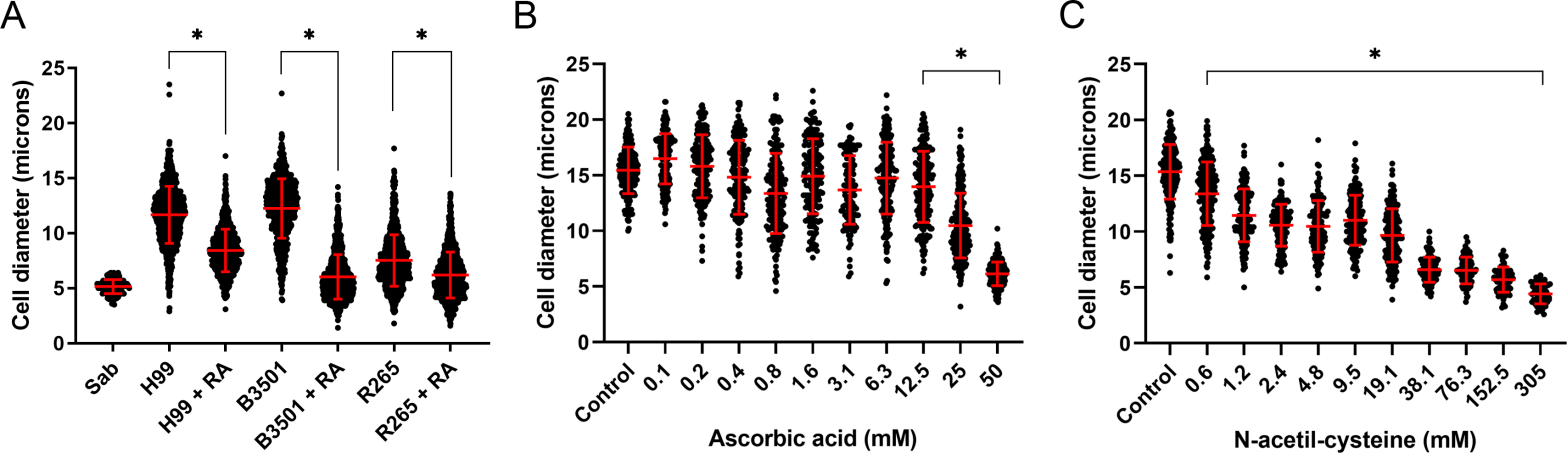
Effect of retinoic acid and other antioxidants in titan cell formation in different strains. *Cryptococcus neoformans* H99, *Cryptococcus deneoformans* B3501, and *Cryptococcus deuterogatti* R265 strains were induced for titanization with and without 25 µM of retinoic acid (RA). The experiment was performed in triplicate. Asterisks denote statistical differences (*P* < 0.0001, Kruskal-Wallis test), and the red bars denote the average and the standard deviation of the population. (**B and C**) Effect of other antioxidants in TC formation. Cells were incubated in TCM in the presence of different concentrations of ascorbic acid (**B**) and N-acetyl cysteine (**C**). Cell size was then determined after lactofuchsin staining as described in Materials and Methods. Asterisks denote statistical differences (*P* < 0.05, Kruskal-Wallis test).

To confirm that retinoic acid inhibits the titanization process, we performed the experiment not only in *Cryptococcus neoformans* H99 strain but also in *Cryptococcus deneoformans* B3501 and *Cryptococcus deuterogatti* R265 strains. As shown in Fig. 4A, we confirmed that retinoic acid inhibited the formation of titan-like cells in different cryptococcal strains.

### Effect of N-acetyl cysteine and ascorbic acid on TC formation

To examine if other antioxidants had any effect on TC development, we evaluated the effect of ascorbic acid and N-acetyl cysteine. As shown in Fig. 4B and C, both antioxidants had an inhibitory effect on titan-like cell formation, with the effect of N-acetyl cysteine being more pronounced.

### Detection of reactive oxygen species during titan-like cell formation

**Fig 5:**
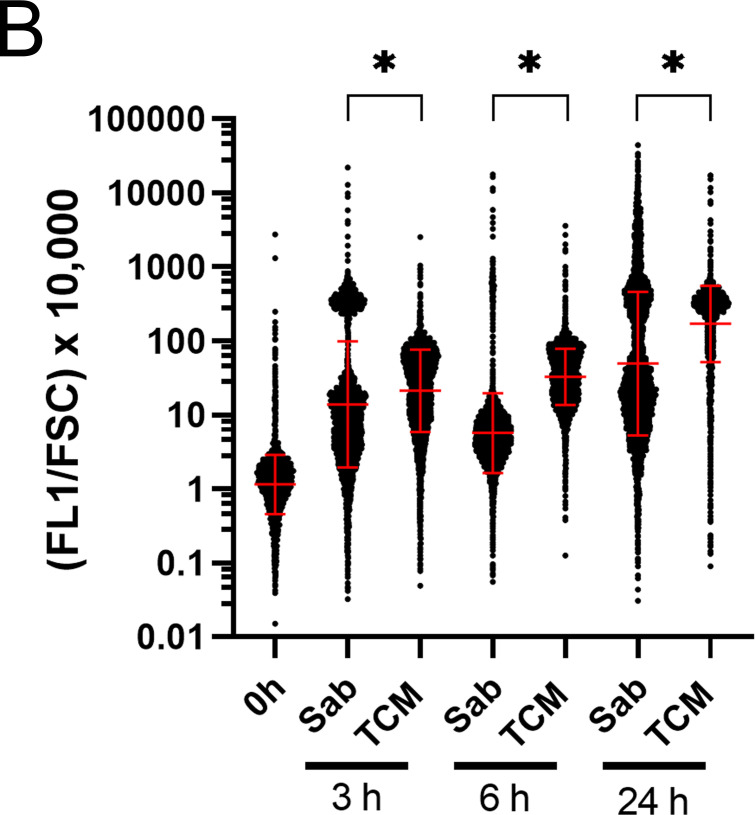
Endogenous ROS detection by DHF during titan-like cell induction. (**A**) Cells from H99 strain were incubated o.n. in Sabouraud liquid medium and then transferred to the same medium (as control of regular cells) or to TCM. ROS were measured at 0, 3, 6, and 24 h after addition of DHF (dark gray histogram) and detection by flow cytometry. A parallel sample without DHF (light gray histogram) was carried out in parallel. A control with Amphotericin B (AmB) 1 µg/mL was added at time 0 h to confirm ROS detection by DHF (data not shown). The experiments were performed twice in two different days. (**B**) Data from cytometry shown in A were exported and processed in Excel to calculate the FL1/FSC ratio (see Materials and Methods). The data were processed and analyzed with GraphPad. The graphs represent the distribution of the individual values obtained from all the cells. (**C**) Effect of retinoic acid (RA) on ROS production during titanization. Titan-like cells were obtained and stained with DHF to measure accumulation of ROS as described in Fig. 5A. Retinoic acid at 25 µM was added at time 0 h to all samples, and ROS production was detected by the addition of 40 µm DHF at times 0, 3, 6, and 24 h. A control without retinoic acid was also measured at each time point. The cytometry data were exported and processed as described in Fig. 5B (FL1/FSC × 10,000), and the geometric mean and standard deviation of the geometric mean are presented in each bar. Asterisk indicates *P* < 0.05.

We observed that some compounds from our list were antioxidants, such as retinoic acid, isotretinoin (or 13-cis retinoic acid), and ebselen. Interestingly, retinoic acid and isotretinoin had almost the same inhibitory profile. As a consequence, we hypothesized that during titanization, an endogenous accumulation of free radicals could trigger a stress signal required for titan-like cell formation. To test this hypothesis, we examined if during this process there was an accumulation of endogenous ROS. We used an ROS-sensitive probe (DHF), which is fluorescent in the presence of free radicals. With this probe, we estimated the production of free radicals in titan cell inducing medium or in media where the cells do not increase in size (Sabouraud). As shown in Fig. 5A, we found that during incubation in TCM, there was a gradual accumulation of ROS in the cells, already noticeable at 3 and 6 h, and all the cells showed a strong fluorescence signal at 24 h. Interestingly, we noticed that in the non-inducing medium Sabouraud, there was a small population of cells that accumulated a significant amount of ROS. This population was not observed when the cells were incubated in the same conditions but without CO_2_ (result not shown), indicating that CO_2_ might generate a stress condition in a subpopulation of cells. After 24 h of incubation in non-inducing conditions, the cells presented a significant fluorescence signal, although it was lower than the peak observed in TCM, indicating that during titan-like cell formation, there is a significant intracellular accumulation of ROS.

To discard that the high amount of ROS in TCM was due to the larger size of the cells, we normalized the fluorescence intensity generated by ROS (FL1) by the size of each cell (FSC parameter). As shown in Fig. 5B, the amount of ROS was still significantly higher in TCM.

We next investigated if the addition of retinoic acid had any effect on the accumulation of ROS in cells incubated in TCM. As shown in Fig. 5C, the addition of the antioxidant reduced the amount of endogenous free radicals accumulated during titanization.

### Mitochondrial membrane potential during titan-like cell formation

**Fig 6:**
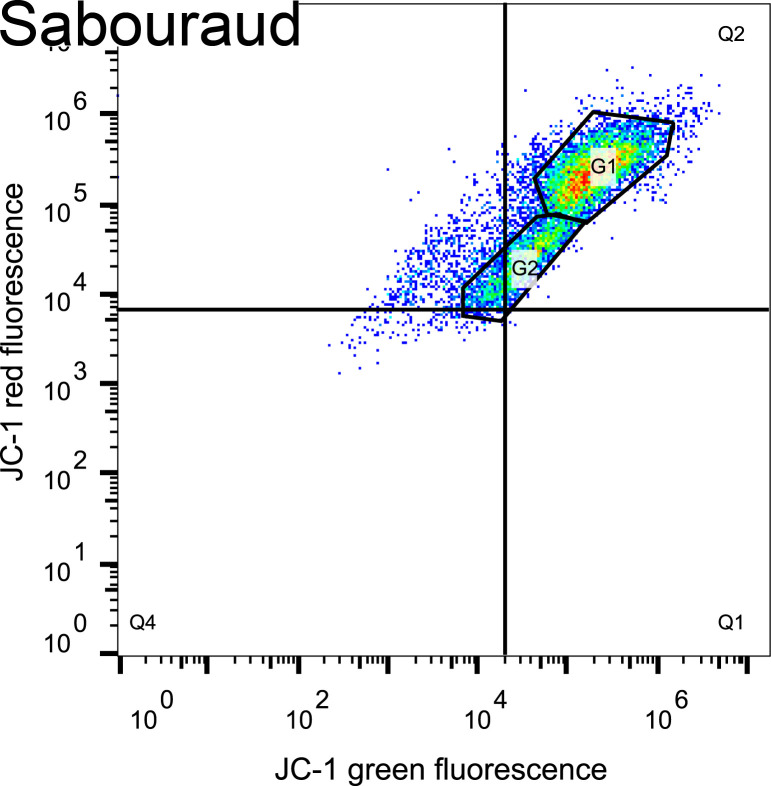
Determination of membrane potential by JC-1 during titan cell-like induction. *C. neoformans* H99 cells were grown o.n. in Sabouraud liquid medium and then transferred to the same medium (as control of regular cells) and to TCM. Mitochondrial membrane potential was measured at 0, 3, 6, and 24 h by flow cytometry after addition of JC-1, and measurement of green (FL-1 channel, x-axis) and red (FL-2 channel, y-axis) fluorescence signals was carried out. The sample of Sabouraud cells at time 0 h was used to determine the quadrants. At some time points, specific gates (denoted by a polygon) were defined for each dot plot to analyze specific cell populations. The experiment was performed in duplicates in two different days.

Since free radicals are mainly produced in mitochondria as subproducts of the respiratory electron transport chain, we evaluated the functionality of this organelle with several complementary approaches. We first measured if there was any variation in the mitochondrial membrane potential using the fluorescent probe JC-1 (see Materials and Methods). This probe can emit both red and green fluorescence, and membrane depolarization is characterized by a decrease in the red/green fluorescence ratio. We added this probe after 0, 3, 6, and 24 h of incubation in TCM and Sabouraud and measured both the red and green fluorescence signals of the cells. We found that the staining was different in both media. In TCM, there was a clear increase of both red and green fluorescence signals of the cells after 3 and 6 h of incubation (Fig. 6). This increase was not noticeable in the non-inducing conditions. The stronger staining suggested that there was an increase in mitochondrial mass during titanization. In addition, we noticed that during titan-like cell formation, there were different populations with different red/green fluorescence ratios. We analyzed these populations using different gates, and we found that there was a partial depolarization of the mitochondria in both conditions (see Table 2). Control cells incubated in Sabouraud medium had a similar ratio than the cells in TCM from gate 1 after 3 and 6 h of incubation. In contrast, the smaller population of cells from gate 2 had a lower ratio. After 24 h of incubation, the ratio of the fluorescence was higher in titan-like cells than in those cells cultivated in Sabouraud medium, indicating that in these titan-like cells, there is not only more mitochondrial mass but also a higher activity than in cells of regular size.

**TABLE 2:** Red/green fluorescence intensity ratios after JC-1 staining during titan-like cell formation^*a*^

	JC-1 red/green fluorescence ratio	
	Sabouraud	TCM
0 h	**1.96**	**1.96**
3 h	**1.42**	**1.35** (*G1, 1.60*) (*G2, 0.42*)
6 h	**1**	**0.62** (*G1, 0.91*) (*G2, 0.25*)
24 h	**0.58** (*G1, 0.50*) (*G2, 0.58*)	**0.92** (*G1, 1.48*) (*G2, 0.97*)

^*a*^ The fluorescence intensity from cells from the cytometry experiment in Fig. 6 was analyzed in Excel and GraphPad, and the ratio of the red/green fluorescence intensity was obtained. We calculated the geometric mean of the whole population (numbers in bold) and of the specific gates (G1 and G2) defined in the graph (numbers in italics).

### MitoTracker staining

**Fig 7:**
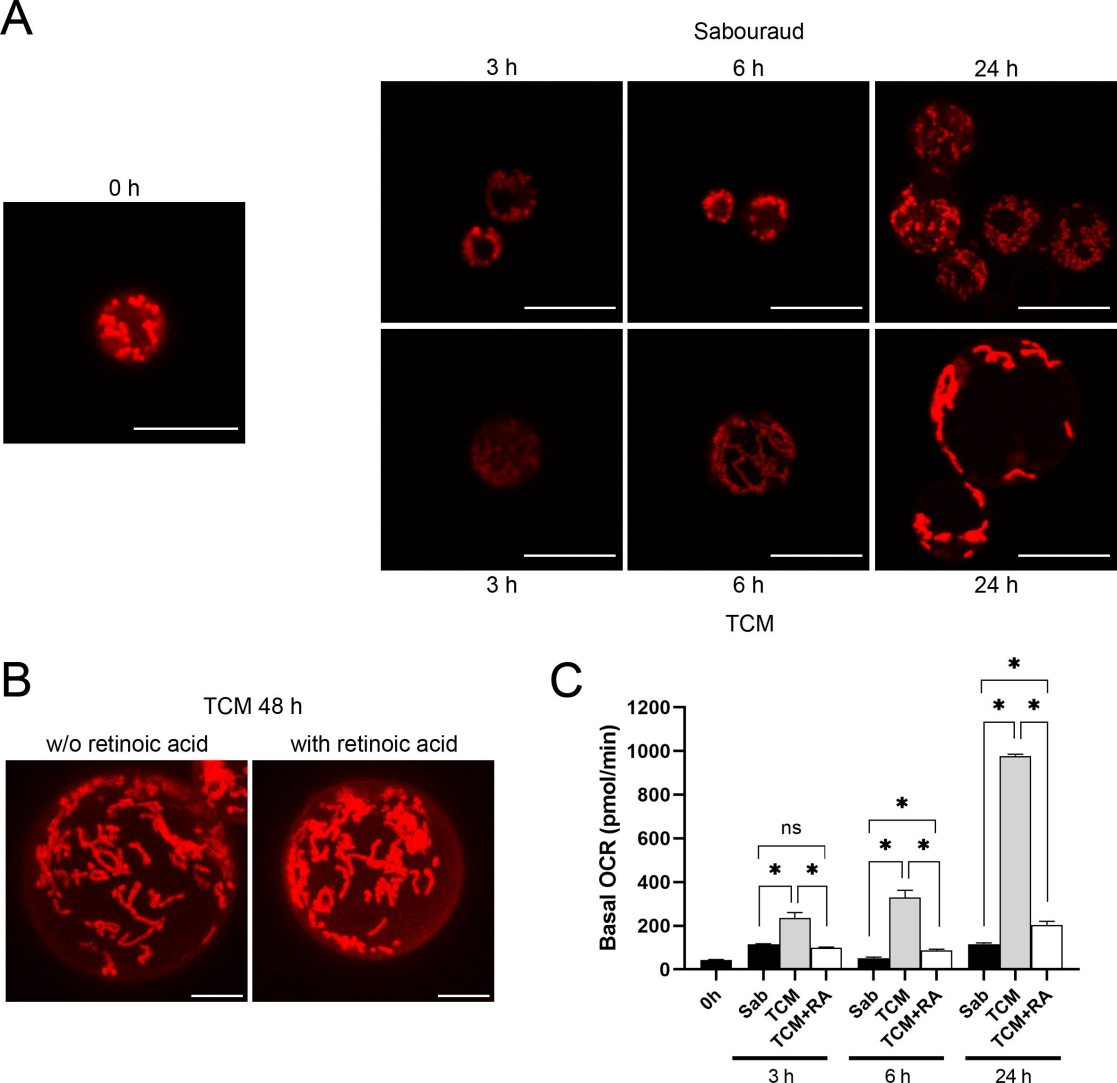
Mitochondrial morphology of *C. neoformans* with MitoTracker Red CMXRos probe. (**A**) Mitochondrial organization was observed by MitoTracker staining (see Materials and Methods). Cells were cultivated in Sabouraud or TCM as described above, and MitoTracker was added at each time point (0, 3, 6, and 24 h). Fluorescence pattern was observed in a Stellaris confocal microscope (upper row, cells incubated in Sabouraud; lower row, cells incubated in TCM). Scale bar in every panel corresponds to 10 µm. (**B**) Effect of retinoic acid (RA) on mitochondrial morphology of titan-like cells. Titanization was induced by incubation o.n. in TCM, and then, titan-like cells were treated with retinoic acid (25 µM) for another 24 h (total incubation time in TCM of 48 h). Then, MitoTracker staining and confocal images were obtained as described in Materials and Methods. Scale bar represents 5 µm. (**C**) Basal oxygen consumption rates (OCR) of titan-like cells, titan-like cells in the presence of 25 µM retinoic acid, and regular cells were measured by a Seahorse XFe24 Analyzer. Different time points were measured (0, 3, 6, and 24 h). Error bars indicate the standard deviation. Experiments were performed in three biological replicates, and one representative replicate is shown. Asterisk indicates *P* < 0.05 (analysis of variance test).

To visualize mitochondrial network organization during titanization, we used MitoTracker dye. In *C. neoformans*, mitochondria can accumulate in a fragmented, tubular, or diffuse pattern (50). Cells incubated in Sabouraud medium provided mainly a fragmented pattern after MitoTracker staining. In contrast, during titan-like cell development, the mitochondria adopted mainly a clear tubular pattern (Fig. 7A).

We next tested if retinoic acid affected the morphology of mitochondria in already formed titan-like cells. As shown in Fig. 7B, we found that retinoic acid did not affect the morphology of mitochondria in TC, indicating that this compound exerted an inhibitory effect during the initial development of these cells.

### Measurement of mitochondrial respiration during TC formation

To further investigate the role of mitochondria during TC formation, we investigated if there was any difference in the respiration rate during this process. For this process, we measured the oxygen consumption rate using a Seahorse XFe24 Analyzer (see Materials and Methods). As we can see in Fig. 7C, the oxygen consumption rate was significantly higher during TC formation compared to cells incubated in non-inducing conditions. Interestingly, addition of retinoic acid reduced the respiration rate during TC development.

### Electron microscopy of the mitochondria

**Fig 8:**
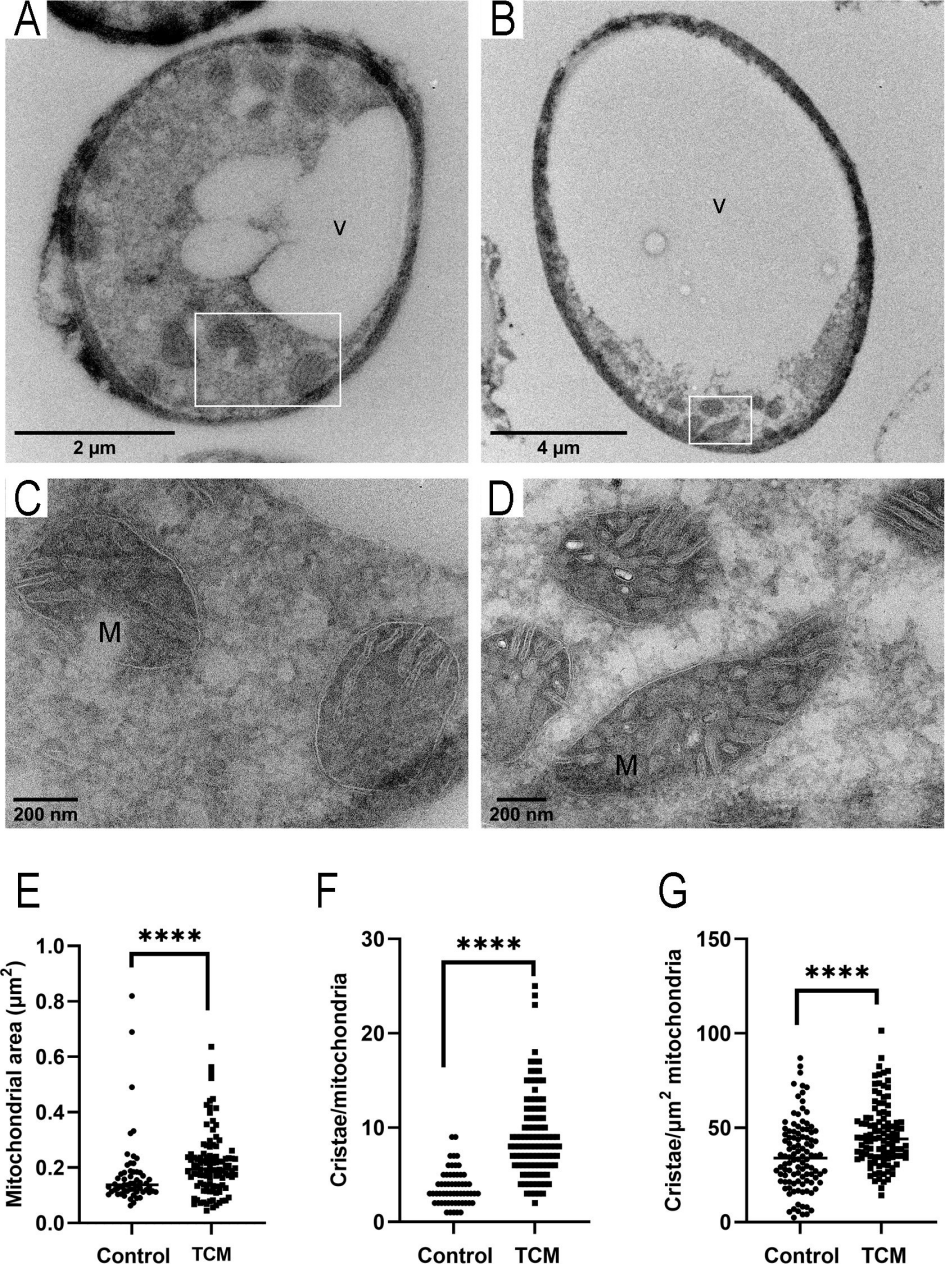
Mitochondrial morphology by transmission electron microscopy. (**A**) Overview image of a regular cell incubated in Sabouraud liquid medium. (**B**) Overview image of a titan-like cell cultivated in TCM. White square shows the mitochondria, shown in C and D, respectively. Arrows indicate cell wall detachment. M, mitochondria; V, vacuole. (**C and D**) Higher magnification images of the morphology of mitochondria in regular cells in Sabouraud (**C**) and titan-like cells (**D**). The area of each mitochondrion (**E**), the number of cristae per mitochondrion (**F**), and number of cristae per square micrometer of mitochondrion (**G**) were quantified and shown in dot plot graphs. Asterisks show statistical differences (*P* < 0.0001, non-parametric Mann-Whitney *t*-test).

Due to the physiological and morphological changes of mitochondria observed by light microscopy, we performed transmission electron microscopy and compared the ultrastructure of this organelle in titan-like cells and regular cells. As shown in Fig. 8, titan-like cells presented a large vacuole, which has already been described (36). We also observed that sample preparation for the analysis of intracellular organelles resulted in partial detachment of cell wall and capsular components (Fig. 8A and B). In contrast, the morphology of the mitochondria was well preserved (Fig. 8C and D). We found that the mitochondria of titan-like cells presented a significantly larger size than those from control cells. Furthermore, there was a striking difference in the number of visible cristae inside the mitochondria. In particular, the matrix of the mitochondria of titan-like cells was compacted with cristae, which was not observed in control cells. In addition, the cristae of control cells had regular spacing between their membranes, whereas the cristae in the titan-like cells showed a swollen, more irregular organization. We quantified the size of the mitochondria and the number of cristae. The average size of the mitochondria from titan-like cells was around 1.2 larger than those from control cells, but the number of cristae and cristae per square micrometer was almost doubled in titan-like cells (Fig. 8E through G; Table 3).

**TABLE 3:** Differences in the mitochondria of cells cultured in Sabouraud medium and TCM^*a*^

	Sabouraud	TCM	Ratio (TCM/Sab)	*P*-value
Mitochondrial area (µm^2^)	0.175 ± 0.131	0.213 ± 0.114	1.22	<0.0001
No. of cristae/mitochondria	3.7 ± 1.9	9.1 ± 4.5	2.5	<0.0001
No. of cristae/mitochondrial area (µm^2^)	25.6 ± 13.9	46.3 ± 16.8	1.8	<0.0001

^*a*^ The size and the number of cristae of the mitochondria identified by electron microscopy was quantified (*n* > 40 for each condition), and the average ± standard deviation of each parameter is shown in the table. Statistical differences were assessed using the Kruskal-Wallis test with Dunn’s post-test for multiple comparisons.

## DISCUSSION

To obtain new insights about the processes involved in titan cell development, we have used a different approach in which we identified the drugs that inhibit this process. For this purpose, we performed a screening using an off-patent library. Since many of the compounds have defined structure and target, we hypothesized that this approach could provide insights on the molecular mechanisms that are required to produce titan cells. Furthermore, drug repurposing has allowed to identify a large number of drugs with antifungal properties (48, 49, 51–59).

During the standardization of the protocol, we observed that subinhibitory concentrations of DMSO (0.5%–1%) partially blocked the induction of titan cells without affecting cell viability or cell division. This solvent can alter the non-covalent bonds that attach the fibers of the capsule (60–62), so it is possible that titan cell formation requires an intact capsule structure to attach new polysaccharide fibers, although we cannot discard that DMSO induces other cell alterations that interfere with this process.

To carry out our screening, we designed a method based on fluorescence as it offers significant advances to perform automatic image analysis. We took advantage of the unspecific binding of the dye lactofuchsin to the surface of fungi. When we compared the binding of this dye with the classical India ink staining, we observed that the size of the cells measured with lactofuchsin was slightly smaller than the one measured with India ink. However, even with this limitation, lactofuchsin staining was still useful to differentiate cells of different size, in particular those incubated in Sabouraud and in the inducing medium TCM. This method offered several advantages, such as fluorescence intensity and low cost.

We identified around 100 inhibitory compounds. Some of them had already been identified in the literature as drugs with antifungal activity against *C. neoformans*. Still, we found more than 60 compounds that selectively inhibited titan cell development. The diversity of compounds that we identified reflects that the induction of TC depends on the induction of multiple and overlapping mechanisms and pathways, which is in agreement with previous findings (41–43). Some of the drugs, such as ebselen, had a fungicidal effect at both high and low concentrations, which has already been described in the literature (63). However, for the rest of the tested compounds, we found a range of inhibitory concentrations that did not correlate with cell killing, suggesting a potential and specific role in TC formation. For this reason, an examination of the potential targets of these compounds could reveal new elements involved in this process.

Mitoxantrone is an antitumoral that inhibits the activity of topoisomerase II and affects DNA replication. In addition, this compound inhibits the activity of a mitochondrial calcium importer (64), which has a putative homolog in *C. neoformans* (CNAG_00107), suggesting that mitoxantrone could also interfere through alteration of mitochondrial activity. In agreement, several of the identified compounds also have effects in this organelle, such as antimycin A (inhibitor of complex III) or pentamidine, which is an antiparasitic that inhibits mitochondrial topoisomerases and causes a reduction in ATP concentration (65–67).

One striking and unexpected finding of our work was that several compounds that act as antioxidants inhibited the formation of titan cells in *C. neoformans*. This was the case with retinoic acid and its isoform isotriteonin and with N-acetyl cysteine and ascorbic acid. Based on this result, we suggested that ROS are required to induce this process. ROS are mainly produced in the mitochondria, so we argued that the activity of this organelle was increased during TC formation. We found that there was a significant increase in respiration and JC-1 staining; a change in mitochondrial organization to a tubular, network-like pattern; and an increase in size and in the number of intramitochondrial cristae. The fact that the increase in ROS occurs at very early times in TCM before other mitochondrial changes (such as the tubular pattern after MitoTracker staining) suggests that ROS might be an early signal to induce TC. It has been described that a tubular organization of the mitochondria is achieved after fusion of this organelle and is associated with resistance to stress conditions and virulence (50, 68, 69). Altogether, our data indicate that TC formation is associated with noticeable changes in mitochondrial activity with a subsequent production of ROS and that this increase in free radicals is required to increase cell size in *C. neoformans*.

ROS concentration in the cell is determined by the balance between its synthesis and detoxification by the antioxidant mechanisms of the cell. Titan cell production requires a significant synthesis of new cellular and capsular components; therefore, the cells also need to increase their mitochondrial activity to produce the energy required for these processes. The finding that some compounds that alter the mitochondrial activity (i.e., antimycin) interfere with TC formation supports this idea.

It could be argued that during TC formation, there is an increase in the number of mitochondria in the cells. However, we believed that our results are not explained in this way. Determining the exact number of mitochondria in the cells is technically very challenging because they cannot be estimated from the transmission electron microscopy (TEM) or MitoTracker images. Furthermore, it has been suggested that the presence of a large vacuole in TC that occupies most of the cytoplasmic volume prevents the cell from investing a significant amount of energy in synthetizing new cellular components, such as mitochondria, endoplasmic reticulum (ER), etc. For these reasons, we argued that the increase in mitochondrial activity is the most probable explanation that supports the increase in intracellular ROS and mitochondrial activity and respiration.

It cannot be discarded that the stress caused by the limitation of nutrients induces a stress signal that leads to ROS accumulation. In this sense, many fungi adapt their morphology between filaments, hyphae, and pseudohyphae, and these changes are normally a response to a stress situation, such as high temperature, nutrient starvation, or serum. In agreement, titan-like cell formation could represent one of the morphological changes induced in stressful environments.

ROS play multiple roles in the cell depending on their concentration. A high concentration of free radicals can produce cellular damage on DNA, lipids, and proteins, all of which are involved in apoptosis. However, ROS have also been involved in signaling pathways (70–72), with beneficial effects in some conditions and participation in cellular homeostasis. ROS can also induce autophagy (73), which is used by the cell to obtain energy from fungal compartments. In the case of TC, the possibility that ROS induce autophagy to obtain energy to trigger cell growth is challenging and deserves more detailed studies in the future.

Our findings are also in agreement with a recent report that also highlights the importance of reactive nitrogen species and ROS in TC formation (74). These authors demonstrated that treatment of the cells with a superoxide dismutase (SOD) mimetic drug (MnTBAP) can also block titanization, a finding that supports the results presented in our work.

Our results might also provide insights about the mechanism of titan cell formation *in vivo*. During infection, the host elicits several responses and antimicrobial mechanisms, which include the production of ROS by phagocytic cells. It is then possible that this oxidative burst triggers the signaling pathways that induce cryptococcal cell growth and further adaptation to the host environment.

In the context of titan cell appearance in the host, we would also like to highlight that the identification of inhibitory compounds might have therapeutic potential. Titan cells are difficult to eliminate from the lungs and offer a selective advantage to the pathogen to persist during long periods. Inhibition of this process might not facilitate killing of fungal cells by the immune system, but it could increase the activity of fungal drugs and augment the efficacy of antifungal therapy.

In conclusion, our work has provided a significant number of compounds that inhibit the formation of titan-like cells. The future characterization of the mechanisms of action of these compounds by the scientific community will be a great contribution to understanding the molecular mechanisms required for this process and to designing new strategies aimed at decreasing fungal adaptation in the host. The findings that during TC formation, there is a significant increase of mitochondrial activity and accumulation of ROS, together with the inhibitory role of antioxidants in this morphological transition, lead us to establish the hypothesis that ROS are key molecules required for TC development in *C. neoformans*.
